# Heat dissipation in quasi-ballistic single-atom contacts at room temperature

**DOI:** 10.1038/s41598-019-55048-3

**Published:** 2019-12-10

**Authors:** Makusu Tsutsui, Yu-Chang Chen

**Affiliations:** 1 The Institute of Scientific and Industrial Research, Osaka University, 8-1 Mihogaoka, Ibaraki, Osaka 567-0047 Japan; 2 Department of Electrophysics, National Chiao Tung University, 1001 University Road, Hsinchu, 30010 Taiwan; 3 College of Artificial Intelegence, National Chiao Tung University, Tainan, Taiwan

**Keywords:** Applied physics, Nanosensors, Electronic devices

## Abstract

We report on evaluations of local heating in Au single-atom chains at room temperature. We performed onsite thermometry of atomic-scale Au junctions under applied sinusoidal voltage of variable amplitudes. The AC approach enabled to preclude electromigration effects for characterizing the influence of energy dissipations on the lifetime. We elucidated nonlinear increase in the effective temperature of the current-carrying single-atom chains with the voltage amplitudes, which was attributed to subtle interplay between electron-phonon scattering and electron-mediated thermal transport in the quasi-ballistic conductor. We also found that only 0.2% of the electric power contributed to local heating while the majority was consumed at the diffusive bank. The present findings can be used for thermal management of future integrated nanoelectronics.

## Introduction

Heat dissipation in nanoscale objects is a fundamental issue in nanoelectronics^1–5^ wherein large current density in the nanostructure under applied biases brings relatively high charge scattering rates^6,7^ thereby inducing significant local heating and concomitant device failures. Atomic-scale metal junctions have been used as a useful model to study the self-heating mechanism in nanoelectronics building blocks^8,9^. Recently, significant progress has been made in this field by the advent of thermometer-embedded mechanical break junctions^8,10^ that allowed direct assessment of temperature change associated with energy dissipation in the current-carrying quasi-ballistic system. However, it has been still technically difficult to place the thermal probe in close proximity to the nanoscale contact forcing one to estimate the actual local temperature from that recorded at more than several tens of nanometers away from it^8^. In contrast, analysis of the natural lifetime of atomic contacts has proven useful for experimentally addressing a change in the local contact effective temperature^11–13^. This onsite nanoscale thermometry led to findings that quasi-ballistic nature of electron transport in the one-dimensional quantum system under DC bias voltage can give rise to significant increase in the effective temperature of the structure via local ionic heating only when the surrounding temperature is far below the Debye temperature^14–17^. On the other hand, effects of the local heating at room temperature are largely unknown despite the direct relevance to the nanoelectronics applications due to significant instability of the Au nanocontacts by the current-induced forces that critically hinders the evaluations of energy dissipation effects on the junction temperature^13,17^. In contrast, we herein report on observations of local heating in Au single-atom junctions at room temperature where we fed AC electrical power to minimize the electromigration-like contributions. As the dissipation is anticipated to involve heat conduction to the bulk, we performed ab-initio calculations to estimate the nano-junction thermal conductivity that enabled derivation of an analytical expression of the voltage-dependent single-atom contact effective temperature.

## Results and Discussions

### Break junction experiments under AC field

**Figure 1:**
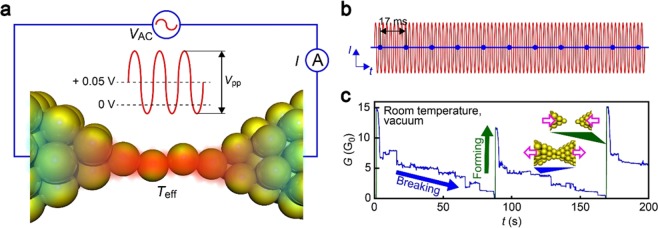
AC break-junction measurements. (**a**) Schematic illustration depicting the measurement circuit. Sinusoidal voltage of frequency 1 MHz and amplitude *V*_pp_ with +0.05 V offset was applied to a Au nano-junction. (**b**) DC component of the output current was recorded by setting 17 ms integration time of a digital multimeter. (**c**) Break-junction experiments using a lithographed mechanically-controllable Au break-junction. Fine-manipulation of the substrate bending via a piezo-motion control enabled formation of Au single-atom contacts that undergo spontaneous breakdown at room temperature in vacuum.

We used a lithographically-defined mechanically-controllable Au break junction^18^ to form a single-atom chain and estimate its stability under the applied sinusoidal voltage of amplitude *V*_pp_ at frequency *F* with DC offset of 0.05 V at room temperature in vacuum (Fig. 1a; see also Figs. S1–S3). Here, we set the integration time of the current sensing system to be much longer than 1/*F* so that we record only DC components, which is defined by the voltage offset, of the output current (Fig. 1b). In this way, we could implement the repetitive junction formation/breakdown by a piezo-control of the substrate bending (Fig. 1c showing a partial conductance trace under AC voltage of *V*_pp_ = 0.2 V and *F* = 1 MHz). During breaking, a special care was taken to minimize the influence of external force on atomic contact stability. For this, we finely-manipulated the junction stretching speed via conductance feedback control of the piezo-actuator motions to let the contact break spontaneously by thermal energy (see Methods for detail). We observed the junction conductance *G* to decrease in a step-wise manner reflecting the discrete atom arrangement processes occurring in the Au junction under the external tensile forces^19–21^. After *G* eventually dropped to zero, we swiftly retracted the piezo-actuator to reconnect the junction to be *G* > 10 G_0_ where G_0_ = 2*e*^2^/*h* is the quantum conductance with *e* and *h* being the electron charge and Plank constant, respectively (Fig. 1c).

### Stability estimation of Au single-atom chains

**Figure 2:**
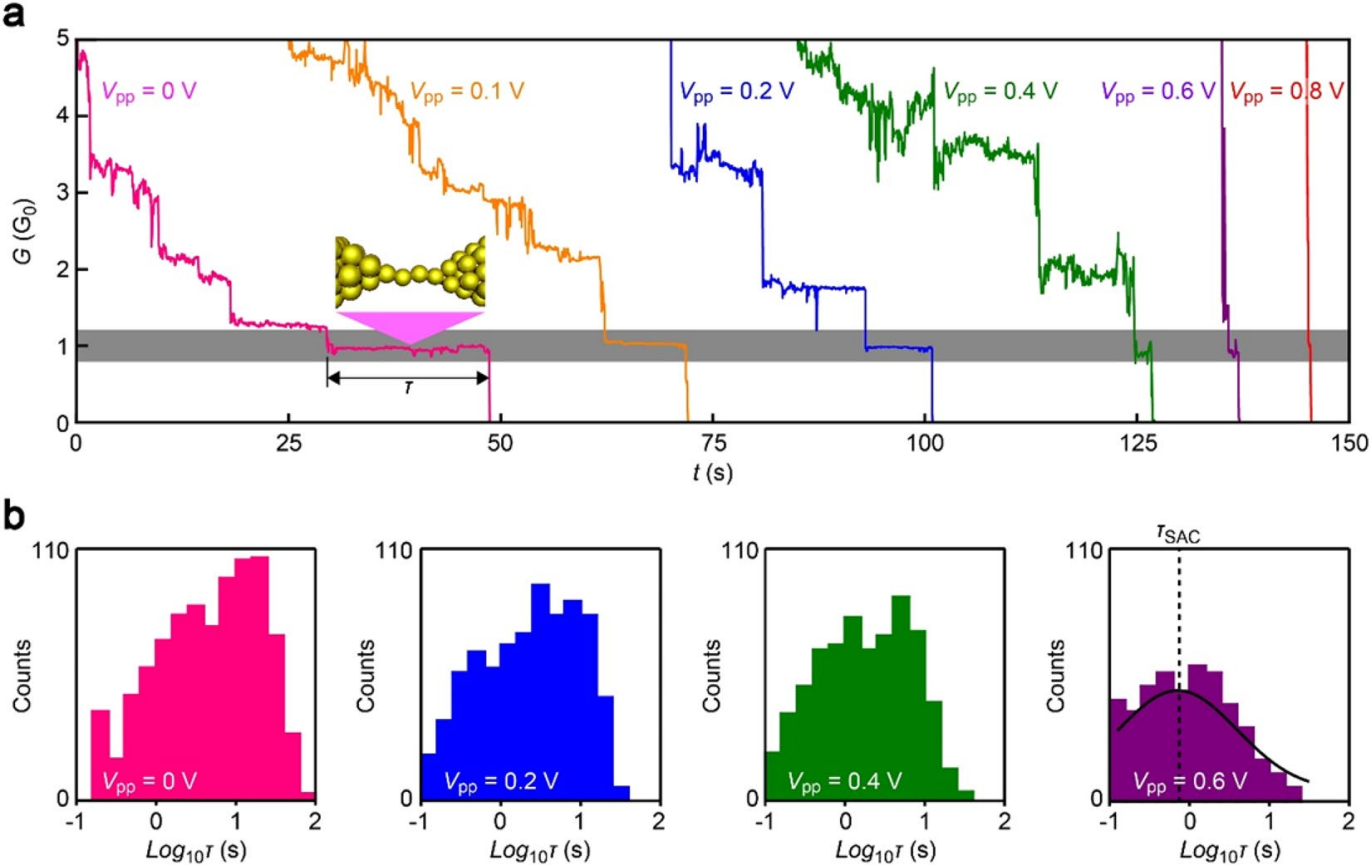
Conductance traces. (**a**) Conductance (*G*) versus time (*t*) curves recorded during junction breaking under various *V*_pp_ conditions. *τ* indicates the length of plateaus appeared at around 1 G_0_, where G_0_ is the conductance quantum. Grey region denotes the range of conductance used to extract *τ*. Junction stretching was ceased at *G* < 10 G_0_ so as to have the single-atom contacts break spontaneously via thermal energy. Color coding indicates *V*_pp_ conditions. (**b**) Log_10_*τ* histograms. Solid curve is a Gaussian fit defining the peak value *τ*_SAC_.

Elongation of Au nano-junctions led to frequent observations of a long plateau at around 1 G_0_. This is a well-known feature reflecting formation of Au single-atom chain having one fully-open channel for electron transmission^19,20^. The duration *τ*_SAC_ for *G* kept at 1 G_0_ can therefore be regarded as lifetime of the monoatomic conductor^11–13^, which was found to become shorter with increasing *V*_pp_ suggesting prominent influence of the AC bias on the contact stability (Fig. 2a).

### Statistical distributions of single-atom contact lifetime

In order for quantitative analyses of the AC-contribution to the single-atom contact durability, we statistically evaluated the change in the lifetime with *V*_pp_ by building *τ*_SAC_ histograms in log-scale through extracting the time period where *G* stayed in a range of 0.8 G_0_ to 1.2 G_0_ from each of the 1000 conductance traces measured (Fig. 2b). The distributions revealed wide variations in the junction fracture time. Here, it is noted that we imposed negligible displacements on the junctions when *G* decreased below 10 G_0_. Because the atomic contacts are metastable structures, they undergo spontaneous breakdown even only by the thermal energy under the external force-free conditions^22,23^. The contact breakdown is thus a thermally-activated stochastic process occurring at random time under given probability defined by the temperature and the barrier energy *E*_B_; when more (less) stable Au contact structure was formed, it tends to be hold for longer (shorter) time^24^. The wide distributions in the lifetime are therefore interpreted as denoting variations in *E*_B_ due to random nature of the junction deformations that create Au single-atom contacts of different configurations in the every breaking trials, and hence different stability.

### AC-bias-induced contact instability

**Figure 3:**
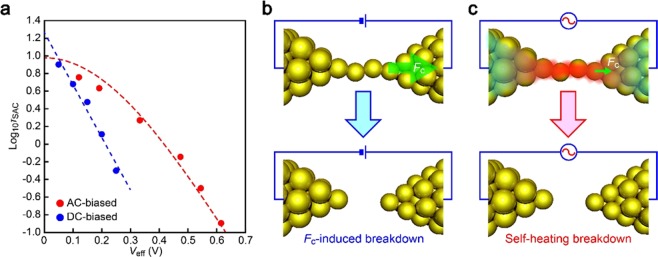
High-field instability of single-atom contacts. (**a**) Semi-logarithmic plots of *τ*_SAC_ as a function of the effective voltage *V*_eff_ biased on Au single-atom contacts. Blue and red circles are the results of DC- and AC-bias measurements, respectively. Blue dashed line is a linear fit to the results obtained under DC bias voltage. Red dashed curve is the lifetime calculated by inserting *T*_eff_ = (*T*_0_^2^ + *αV*_eff_^2^)^1/2^ into the Arrhenius equation of the contact lifetime with the coefficient *α* = 280 K^2^/V^2^. (**b**,**c**) Schematic models depicting breakdown modes of DC- (**b**) and AC-biased contacts (**c**). Predominant effects of the current induced force *F*_c_ largely determine the lifetime of DC-biased contacts while *F*_c_ is small under the AC field that leads the junction to fracture via local heating.

AC voltage effects on the single-atom contact stability were investigated through extracting and plotting the peak values *τ*_SAC_ in the lifetime histograms by Gaussian fits to the distributions against *V*_pp_ (Fig. 3a). The result displayed nonlinear decrease in the logarithmic lifetime against the effective voltage *V*_eff_ = *V*_pp_/√2 relevant to the energy dissipation. This is to be compared to the case of DC-biased single-atom chains whose log_10_*τ*_SAC_ (obtained through the same methods but with direct voltage *V*_dc_ = *V*_eff_) gets rapidly shorter in a linear fashion with the voltage (Fig. 3a).

### Underlying physics of high-field contact instability

The marked difference in the *V*_eff_ dependence of *τ*_p_ between the AC and DC bias approaches can be rationally explained by contributions of current-induced forces. Under direct voltage, huge current density generates charge imbalance that yields local force to dispart the interatomic distance in the atomic wire^25^. This electromigration-like phenomenon was reported to shorten the lifetime as

$$\tau ={{f}_{0}}^{-1}\exp [\,-\,({E}_{{\rm{B}}}-\beta {V}_{{\rm{dc}}})/{k}_{{\rm{B}}}T],$$

where the Arrhenius expression describes the thermally activated spontaneous breakdown of the atomic chains under the thermal energy *k*_B_*T* and the attempt frequency *f*_0_ = 3.5 THz^26^ with the Boltzmann factor *k*_B_ and the temperature *T*^23^. In the equation, the current-induced force enters as *βV*_dc_ that acts to reduce the barrier energy against the contact rapture *E*_B_. Indeed, the linear Log*τ*_p_ versus *V*_eff_ characteristics of the DC-biased Au single-atom contacts manifests the prevailing effect of the field-mediated contact embrittlement^25^ with *β* = 0.35 eV/V and *E*_B_ = 0.80 eV as deduced by least-square fitting (Fig. 3b; see Fig. S4 for the *V*_DC_-dependent lifetime histograms). Furthermore, it in turn suggests the relatively weak change in *T* via local heating due presumably to efficient heat conduction to the bulk^15^.

Whereas electromigration is a crucial factor limiting the contact lifetime of DC-biased self-breaking Au atomic contacts, the situation is anticipated to be completely different under the AC field since the back-and-forth charge flow should effectively cancel the net wind force^27^. Therefore, *βV*_dc_ is expected to be small irrespective of *V*_pp_ having only the offset contribution amounting *V*_dc_ = 0.05 V. What remains is local heating by AC-derived energy dissipation as a possible cause of the nonlinear Log*τ*_p_ − *V*_eff_ dependence (Fig. 3c).

### Room temperature heat dissipation at single-atom contacts

**Figure 4:**
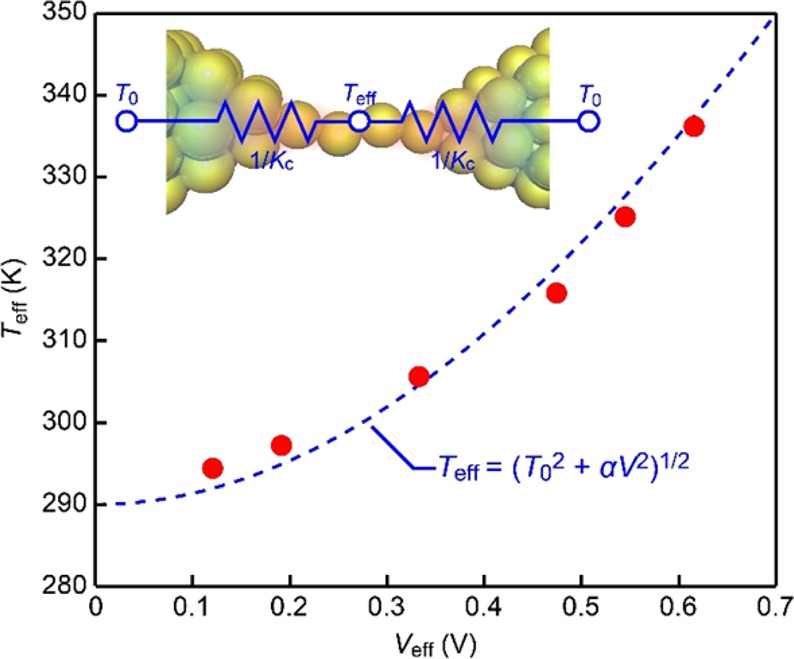
Heat dissipation in Au single-atom contacts at room temperature. The effective temperature *T*_eff_ of AC-biased single-atom contacts plotted with respect to *V*_eff_. Dashed line is fitting with a steady-state model of local heat dissipation. Inset shows an equivalent circuit of self-heated Au single-atom chain comprised of a heat source at *T*_eff_ whose both sides connected to two sinks at the ambient temperature *T*_0_ via thermal resistance 1/*K*_c_.

In order to shed light on the local heating in the quasiballistic single-atom contacts at room temperature, we deduced the effective temperature *T*_eff_ at the breaking points from *τ*_p_ of the AC-biased contacts with the constant DC effect. More specifically, using the coefficient *β* and *E*_B_ values obtained in the DC measurements, we back-calculated *T*_eff_ from the Arrhenius expression of the lifetime with *τ* = *τ*_p_. The estimation showed pronounced increase in the contact temperature with *V*_eff_ (Fig. 4). When assuming steady-state heat dissipation, the energy balance can be approximated as

$${P}_{{\rm{c}}}=2{K}_{{\rm{c}}}\Delta T$$

considering transport of energy per time *P*_c_ dissipated at the single-atom contact to the heat sinks via the thermal conductance *K*_c_ along the temperature gradient Δ*T* = *T*_eff_ − *T*_0_ with the ambient temperature *T*_0_ = 290 K (see Fig. 4a inset)^27^. Here, *K*_c_ is anticipated to be not so different from the thermal conductance of the single-atom contact. This is because of the large Au banks with high thermal conductivity directly connected to the atomic chain that serve as heat reservoirs. In other words, the dissipation-derived temperature drop is anticipated to occur mostly at around the atomic contact having high thermal resistance whereas the bulk compartment stays at *T*_0_ and plays minor roles on *P*_c_.

### Thermal conductance carried by electrons and phonons

The heat generated at the single-atom contacts is anticipated to dissipate mainly through the Au banks via thermal conduction. It is thus a prerequisite to know the thermal transport property of the atomic-scale conductor considering the expected intimate roles of heat conduction on *T*_eff_ − *V*_eff_ characteristics. The thermal conductance *K*_*c*_ comprises contributions from electrons and phonons: *K*_*c*_ = *K*_*el*_ + *K*_*ph*_. In low-temperature regime (*T* ≪ *T*_D_), *K*_*ph*_ ∝ *T*^3^ due to quantum statistics of phonon transport. Note that *T*^3^-law is valid only for temperatures well below the Debye temperature *T*_D_. In a high-temperature regime (*T* ≳ *T*_D_), the statistics of phonon is classical and quantum correction is unimportant giving *K*_*ph*_ ≈ *κ*_*ph*,*MD*_ with constant *K*_*ph*,*MD*_^28^. In the intermediate-temperature regime, on the other hand, quantum correction is required^26^ for *K*_*ph*_ given as

$${K}_{ph}={K}_{ph,MD}\frac{d{T}_{MD}}{dT}$$

**Figure 5:**
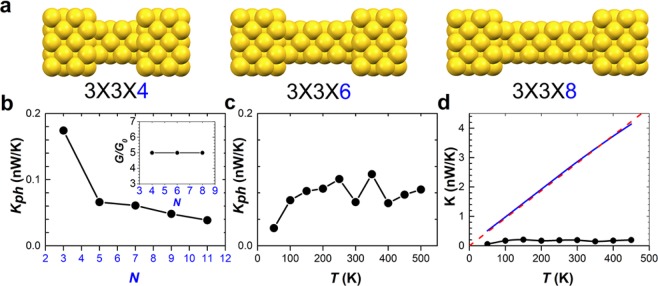
Wiedemann-Franz law & competition between *K*_*el*_ and *K*_*ph*_. (**a**) Schematics of nano-junctions formed by Au nanowires connected to Au(100) electrodes. The Au nanowire, denoted by 3 × 3 × N, has a cross section of 3 × 3 atom layers and a length of *N* atoms layers. (**b**) Phononic thermal conductance *K*_*ph*_ at *T* = 300 K for *N* = 3, 5, 7, 9, 11. Inset shows the electric conductance ≈ 5 G_0_, which is independent of the length of the Au wire. (**c**) *K*_*ph*_ as a function of temperatures for *N* = 4. (**d**) Electronic thermal conductance *K*_*el*_ (blue solid) compared with *K*_*ph*_ (solid circles) as a function of temperatures for *N* = 4. Red-dashed line represents the Wiedemann-Franz law: *K* = *LGT*, where *G* ≈ 5 G_0_.

For metallic junctions, electrons travel between electrodes via resonant tunneling without much efforts such that *K*_*el*_ ≫ *K*_*ph*_. This validates the Wiedemann-Franz law *K*_*c*_ ≈ *LGT*, where *G* is insensitive to temperature and wire length. The above-mentioned properties are illustrated in Fig. 5, where we take 3 × 3 × *N* Au nano-junctions as examples (Fig. 5a). Figure 5b depicts that *K*_*ph*_ decreases as the length of wire increases whereas the dependence of *G* on length is very weak. The Debye temperatures of Au nanowires are around 100 K, which are suppressed by the small diameter compared with *T*_*D*_ ≈ 160 K for bulk gold^29^. As demonstrated in Fig. 5c, *K*_*ph*_ tends to a constant value and shows classical behavior under high-temperature conditions (*T* ≳ *T*_D_ ≈ 100 K). On the other hand, quantum correction becomes important at *T* = 50K in intermediate-temperature regime. As a whole, the theory predicts *K*_*c*_ ≈ *LGT* at the range of *T*_eff_ in the present work due to *K*_*el*_ ≫ *K*_*ph*_ (Fig. 5d), which is also in accordance to recent experiments^30,31^.

### Room temperature local heating mechanism

With *K*_*c*_ ≈ *LGT*, we arrive at expression^32^ *T*_eff_ ~ (*T*_0_^2^ + *αV*_eff_^2^)^0.5^ with *T* = (*T*_eff_ + *T*_0_)/2 that qualitatively agrees with the experimental observation (dashed line in Fig. 4). It is worth noting that the voltage dependence of the room temperature local heating differs from *T*_eff_ ~ √*V*_eff_ observed at 4.2 K^14,15^. The difference can be ascribed to intricate roles of phonons giving rise to distinct temperature dependence of the material thermal conductivity^15,33^.

**Figure 6:**
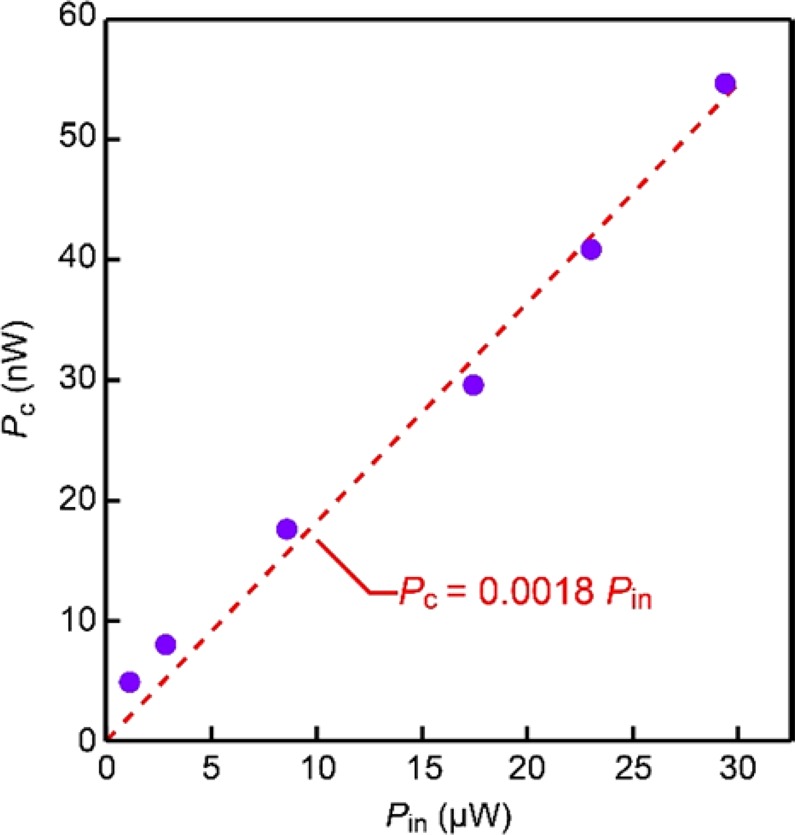
Power consumption at Au single-atom contacts. Relation between the input power *P*_in_ and the energy dissipated at the single-atom contact *P*_c_. Dashed line is a linear fit with zero intercept showing less than 2% of the electric power used in effect for local heating at the quasi-ballistic atomic wire.

More quantitatively, the thermal conductance *K*_SAC_ of the single-atom wire with *G*_SAC_ = 77.5 μS can be acquired by *K*_SAC_ = *LG*_SAC_*T*. Back-calculation of *P*_c_ from *K*_SAC_ suggested that only 0.2% of the μW-level input power was dissipated at the atomic contacts (Fig. 6). This is not surprising as most of the kinetic energy of the field-accelerated electrons should be released at the phonon baths several tens of nm away from the atomic junction as noted by the inelastic mean free path of gold^34^.

## Conclusions

We quantitatively evaluated local heating in a current-carrying quasiballistic conductor at room temperature. AC voltage was used to impose electric power to Au atomic wires, the condition of which is relevant to practical situations of fast switching nanoelectronic devices. We observed nonlinear increase in the effective temperature with the input power suggesting steady-state heat dissipation occurring at the single-atom junctions. Meanwhile, the local temperature was found to increase by only several tens of Kelvins under μW-level input power suggesting only 0.2% of the electric energy being converted to heat at the atomic contacts while the majority is released at the banks by virtue of the quasi-ballistic charge transport in the one-dimensional quantum system. The present finding can be used as a useful guide for establishing heat managements in nanoelectronic circuits.

## Methods

### Fabrications of microfabricated mechanically-controllable break junctions

One side of a phosphor-bronze substrate was coated by 10 μm-thick polyimide through spin-coating and baking. On the polyimide layer, Au micro-electrodes were patterned with a Cr adhesion layer by photolithography followed by radio-frequency magnetron sputtering of metals and lift-off in *N*,*N*-dimethylformamide (Wako) by sonication. Using parts of the microelectrodes as markers, nano-junctions were further delineated that had 100 nm width at the narrowest constriction by electron beam lithography (Elionix). Each junction was designed to overlap with two microelectrodes at the both sides. Subsequently, the sputtering process was implemented to deposit 100 nm thick Au layer on pre-sputtered 5 nm-thick Cr. The substrate was kept in *N*,*N*-dimethylformamide overnight to dissolve the residual resist for lift-off. After fabricating the Au junctions, we exposed the entire substrate surface to isotropic reactive ion etching (RIE-10NR, Samco) with O_2_ etchant gas (see also Fig. S1). This served to completely remove the polyimide underneath the junction so as to make it partially free from the substrate at the narrowed part of length 2 μm (Fig. S2). Finally, the substrate was baked at 200 degrees Celsius for annealing of the sputtered Au structure.

### Conductance measurements

Electrical voltage was applied to a Au junction through two microelectrodes and the output current was measured using a digital multimeter (PXI-4071, National Instruments). DC measurements were performed by using a digital acquisition board (PXI-6281, National Instruments) to feed direct voltage while AC measurements were carried out by applying sinusoidal voltage of frequency *F* = 1 MHz and amplitude *V*_pp_ = 0 to 1.0 V with an offset of 0.05 V using a function generator (33500B, Agilent). The current measurements were exhibited under 17 ms integration time that enabled to exclude the AC components of the output within the *V*_pp_ range tested.

### Break junction experiments

The Au junction substrate was mounted on a sample stage in a three-point bending configuration. The stage was enclosed in a chamber, which was then evacuated using a turbo molecular pump to a level below 10^−5^ Torr. After that, the substrate was bent mechanically by moving a pushing rod under a screw mechanism. Meanwhile, the junction conductance was monitored, which showed to decrease gradually upon necking deformation of the Au junction. When the Au contact eventually broke and the conductance dropped to zero, the rod was swiftly retracted to fuse the junction. Later, the pushing rod was carefully moved via a piezo-control (HJPZ-0.15P, Matsusada Precision) to implement repetitive contact breaking/forming under a LabVIEW program. There, the junction stretching speed was controlled by a conductance feedback loop to stop the piezo-motion at the stage where the conductance decreased to below 10 G_0_ in every junction breaking processes. When the contacts ruptured, we reconnected them again by reversing the pushing rod position until the conductance became higher than 10 G_0_. The above processes were repeated for 1000 cycles and recorded the conductance versus time curves during the junction elongation (Fig. S3).

#### Thermal conductance carried by electrons and electric conductance

Nano-junctions formed by Au nanowires sandwiched between Au(100) electrodes are relaxed using the Vienna Ab-Initio Simulation Package (VASP). The nanowire, denoted by 3 × 3 × N, has a cross section of 3 × 3 atom layers and a length of *N*-atom layers, as depicted in Fig. 5a. Nanodcal code is performed to calculate transmission functions [*τ*(*E*)] of optimized structures^35^. Nanodcal is based on non-equilibrium Green’s function combined with density functional theory, where local density approximation is selected for the exchange and correlation potentials. The self-consistent calculations terminate at a criterion of accuracy at 10^−5^ eV. We apply *τ*(*E*) to calculate the electric conductance (*G*) and thermal conductance (*K*_*el*_), respectively^36^:

1 $$G(T)={G}_{0}{L}_{0}(\mu ,T),$$

where ${G}_{0}=\frac{2{e}^{2}}{h}$ is the quantum unit conductance, and the electronic thermal conductance is

2 $${K}_{el}(\mu ,T)=\frac{2}{h}[-\frac{1}{T}\frac{{[{L}_{1}(\mu ,T)]}^{2}}{{L}_{0}(\mu ,T)}+\frac{{L}_{2}(\mu ,T)}{T}],$$

where

3 $${L}_{n}(\mu ,T)=\int dE(-\frac{\partial f(\mu ,T)}{\partial E}){(E-\mu )}^{n}\tau (E),$$

where *f*(*E*, *T*) = 1/(*e*^(E−μ)*k*^_*B*_^*T*^ + 1) is the Fermi-Dirac distribution and μ is the chemical potential. *L*_*n*_(*μ*, *T*) can be expressed as a polynomial of *T* via Sommerfeld expansion, To the lowest order in *T*, we observe that the electronic thermal conductance obeys the Wiedemann-Franz law: *K*_*el*_ = *LGT*, where the Lorenz number *L* = *π*^2^*k*_*B*_^2^/3*e*^2^ ^28,37^.

### Thermal conductance carried by phonons

The phononic thermal current is simulated by Non-equilibrium molecular dynamic (NEMD) simulations. We select *Large-scale Atomic/Molecular Massively Parallel Simulator* (LAMMPS) package to calculate phononic thermal conductance *K*_*ph*_ owing to vibrations of atoms^38–40^. A temperature difference across the 3 × 3 × N Au wire is set to generate the phononic thermal current. The electrodes are modeled as hot and low temperature reservoirs with temperatures controlled by the Nose-Hoover thermostat method using LAMMPS code. Both electrodes have in total 256 Au atoms. The temperature of the hot electrode is set 10% higher, whereas the temperature of the cold electrode is set 10% lower than the average temperature of the system. We select Embeddedatom Method (EAM) for the force field. The NEMD simulations are performed in a temperature of the system ranging from 50 K to 500 K. The NEMD simulations are carried out with a time step of 0.5 fs for a time period of 10 ns. The classical phononic thermal conductance *κ*_*ph*,*MD*_ is obtained by computing the heat flux due to the temperature gradient^39–41^. We also apply quantum correction for *K*_*ph*,*MD*_ to obtain the corrected phonon’s thermal conductance *K*_*ph*_^41^,

4 $${K}_{ph}={K}_{ph,MD}\frac{d{T}_{MD}}{dT},$$

where

5 $$3N{k}_{B}{T}_{MD}=\int d\omega {D}_{ph}(\omega )\hslash \omega (n(\omega ,T)+1/2),$$

where *D*_*ph*_(*ω*) is the phonon density of states calculated from the Fourier transform of the atoms’ velocity autocorrelation functions. The quantum statistics is given by the Bose-Einstein distribution: *n*(*ω*, *T*) = 1/(*e*^*ℏω*/*k*^_*B*_^*T*^ − 1), and $\hslash$*ω*/2 the zero-point energy.

## Supplementary information

Supplementary Information

## References

[1] Ball P (2012). Computer engineering: Feeling the heat. Nature.

[2] Pop E (2010). Energy dissipation and transport in nanoscale devices. Nano Res..

[3] Aradhya SV, Venkataraman L (2013). Single-molecule junctions beyond electronic transport. Nat. Nanotechnol..

[4] Comtet J, Laine A, Nigues A, Bocquet L, Siria A (2019). Atomic rheology of gold nanojunctions. Nature.

[5] Luo Z (2019). Structure-property relationships in graphene-based strain and pressure sensors for potential artificial intelligence applications. Sensors.

[6] Chen Y-C, Zwolak M, Di Ventra M (2003). Local heating in nanoscale conductors. Nano Lett..

[7] Dubi Y, Di Ventra M (2011). Colloquium: Heat flow and thermoelectricity in atomic and molecular junctions. Rev. Mod. Phys..

[8] Lee W (2013). Heat dissipation in atomic-scale junctions. Nature.

[9] Tsutsui M, Kawai T, Taniguchi M (2012). Unsymmetrical hot electron heating in quasi-ballistic nanocontacts. Sci. Rep..

[10] Tsutsui M, Morikawa T, Yokota K, Taniguchi M (2018). Remote heat dissipation in atom-sized contacts. Sci. Rep..

[11] Huang Z, Xu B, Chen Y, Di Ventra M, Tao N (2006). Measurement of current-induced local heating in a single molecule junction. Nano Lett..

[12] Huang Z (2007). Local ionic and electron heating in single-molecule junctions. Nat. Nanotechnol..

[13] Tsutsui M, Taniguchi M, Kawai T (2008). Local heating in metal-molecule-metal junctions. Nano Lett..

[14] Tsutsui M, Kurokawa S, Sakai A (2006). Bias-induced local heating in Au atom-sized contacts. Nanotechnology.

[15] Todorov TN (1997). Local heating in ballistic atomic-scale contacts. Philos. Mag. B.

[16] Sperl A, Kroger J, Berndt R (2010). Direct observation of conductance fluctuations of a single-atom tunnelling contact. Phys. Rev. B.

[17] Dundas D, McEniry EJ, Todorov TN (2009). Current-driven atomic waterwheels. Nat. Nanotechnol..

[18] van Ruitenbeek JM (1996). Adjustable nanofabricated atomic size contacts. Rev. Sci. Instrum..

[19] Agrait N, Yeyati AL, van Ruitenbeek JM (2003). Quantum properties of atomic-sized conductors. Phys. Rep..

[20] Yanson, A. I., Rubio-Bollinger, G., van den Brom, H. E., Agrait, N. & van Ruitenbeek, J. M., Formation and manipulation of a metallic wire of single gold atoms. *Nature* **395**, 783–785 (1998).

[21] Ohnishi H, Kondo Y, Takayanagi K (1998). Quantized conductance through individual rows of suspended gold atoms. Nature.

[22] Dreher M (2005). Structure and conductance histogram of atomic-sized Au contacts. Phys. Rev. B.

[23] Tsutsui M, Shoji K, Taniguchi M, Kawai T (2008). Formation and self-breaking mechanism of stable atom-sized junctions. Nano Lett..

[24] Evans E, Ritchie K (1997). Dynamic strength of molecular adhesion bonds. Biophys. J..

[25] Todorov TN, Hoekstra J, Sutton AP (2001). Current-induced embrittlement of atomic wires. Phys. Rev. Lett..

[26] Agrait N, Untiedt C, Rubio-Bollinger G, Vieira S (2002). Electron transport and phonons in atomic wires. Chem. Phys..

[27] Tao J, Liew B-K, Chen JF, Cheung NW, Hu C (1998). Electromigration under time-varying current stress. Microelectron. Reliab..

[28] Amanatidis I, Kao J-Y, Du L-Y, Pao C-W, Chen Y-C (2015). Thermoelectric efficiency of single-molecule junctions: phase diagram constructed from first-principles calculations. J.Phys. Chem. C.

[29] Xiong S (2011). Universal relation for size dependent thermodynamic properties of metallic nanoparticles. Phys. Chem. Chem. Phys..

[30] Cui L (2017). Quantized thermal transport in single-atom junctions. Science.

[31] Mosso N (2017). Heat transport through atomic contacts. Nat. Nanotechnol..

[32] Holm, R. Electric Contacts, *Springer*, Berlin (1967).

[33] Lavasani A, Bulmash D, Sarma SD (2019). Wiedemann-Franz law and Fermi liquids. Phys. Rev. B.

[34] Zheng J, Zhang C, Dickson RM (2004). Highly fluorescent, water soluble, size tunable gold quantum dots. Phys. Rev. Lett..

[35] Taylor J, Guo H, Wang J (2001). Ab initio modeling of quantum transport properties of molecular electronic devices. Phys. Rev. B.

[36] Liu Y-S, Chen Y-R, Chen Y-C (2009). Thermoelectric efficiency in nanojunctions: a comparison between atomic junctions and molecular junctions. ACS Nano.

[37] Kaun C-C, Chen Y-C (2018). Thermoelectric charge and spin current generation in magnetic single-molecule junctions: first-principles calculations. J. Phys. Chem. C.

[38] Mller-Plathe FA (1997). Simple nonequilibrium molecular dynamics method for calculating the thermal conductivity. J. Chem. Phys..

[39] Plimpton S (1995). Fast parallel algorithms for short-range molecular dynamics. J. Comp. Phys.

[40] Lammps. http://lammps.sandia.gov (2010).

[41] Yang N, Zhang G, Li B (2008). Ultralow thermal conductivity of isotope-doped silicon nanowires. Nano Lett..

