# Supplementary material for: Heat dissipation in quasi-ballistic single-atom contacts at room temperature
Source: Sci Rep. 2019 Dec 10;9:18677. doi: 10.1038/s41598-019-55048-3 (PMC6904740; doi:10.1038/s41598-019-55048-3)
Supplement: Supplementary file 1 — Supplementary Information [file 41598_2019_55048_MOESM1_ESM.pdf]

Supplementary Information

Heat dissipation in quasi-ballistic single-atom contacts at room temperature

Makusu Tsutsui and Yu-Chang Chen

The Institute of Scientific and Industrial Research, Osaka University, Ibaraki, Osaka 567-0047, Japan

E mail: tsutsui@sanken.osaka-u.ac.jp

The Supplementary Information includes:

1. Supplementary Figures (Fig. S1-S3)

2. Supplementary references

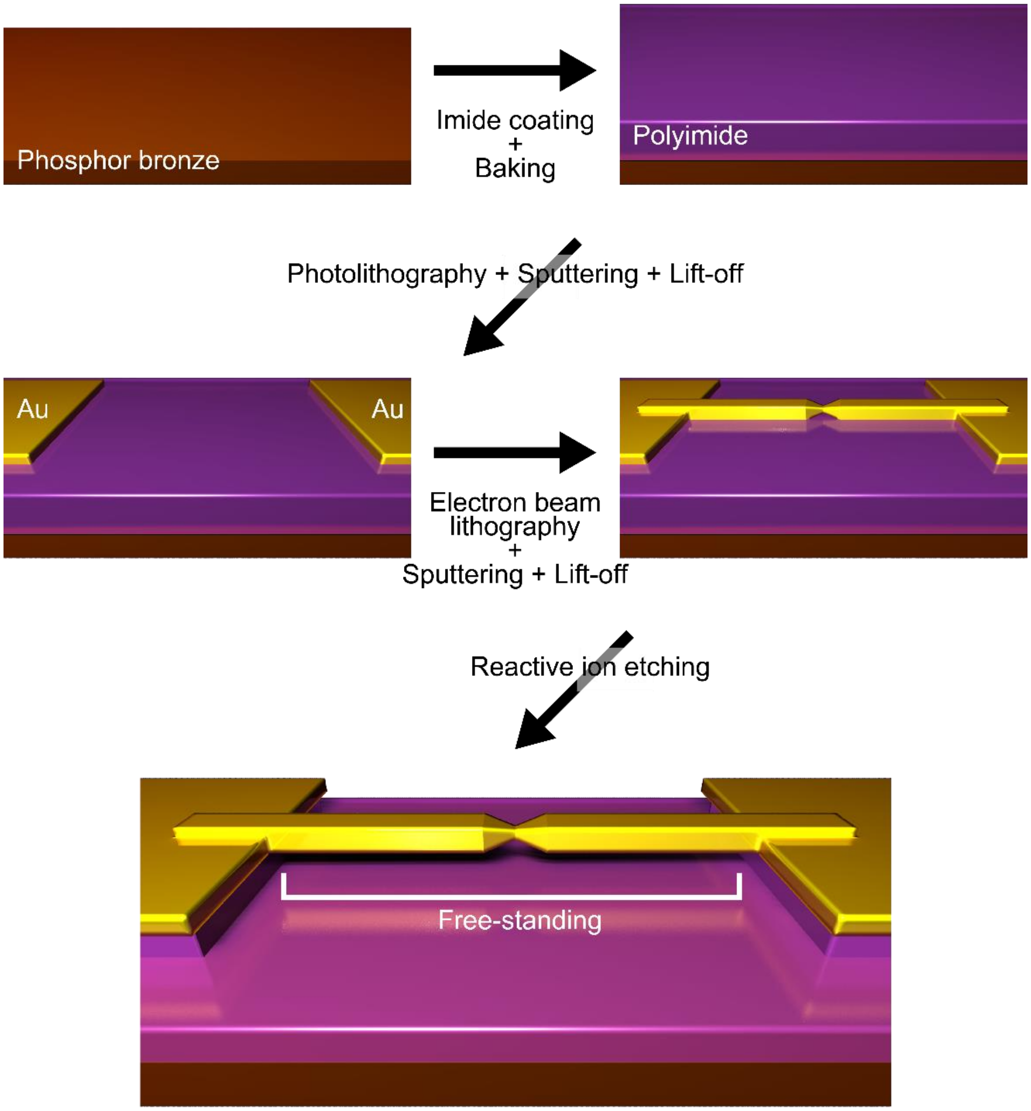

Figure S1. Fabrication processes of lithographically-defined mechanically-controllable break junctions.

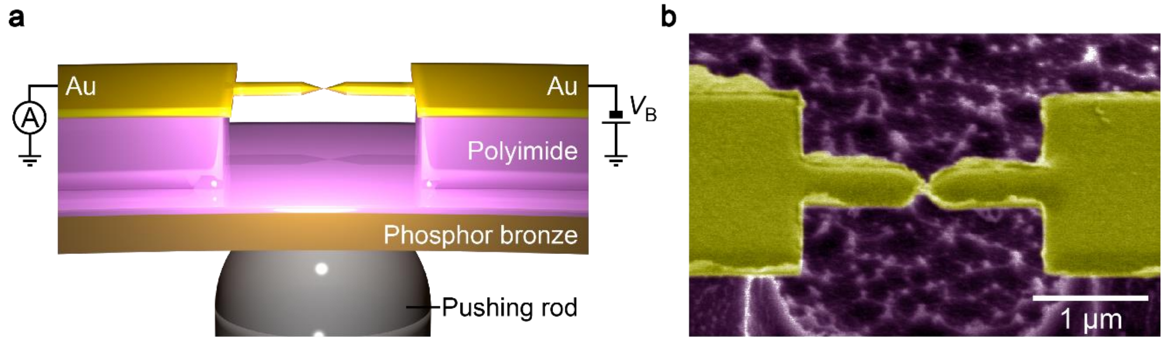

Figure S2. a-b, Schematic (a) and scanning electron micrograph (b) of a micro-fabricated mechanically-controllable break junction (MCBJ). A Au nanowire was formed on a polyimide-coated phosphor-bronze substrate by nano-fabrication processes.[S1] The narrowest part had approximately 100 nm x 100 nm cross-section.

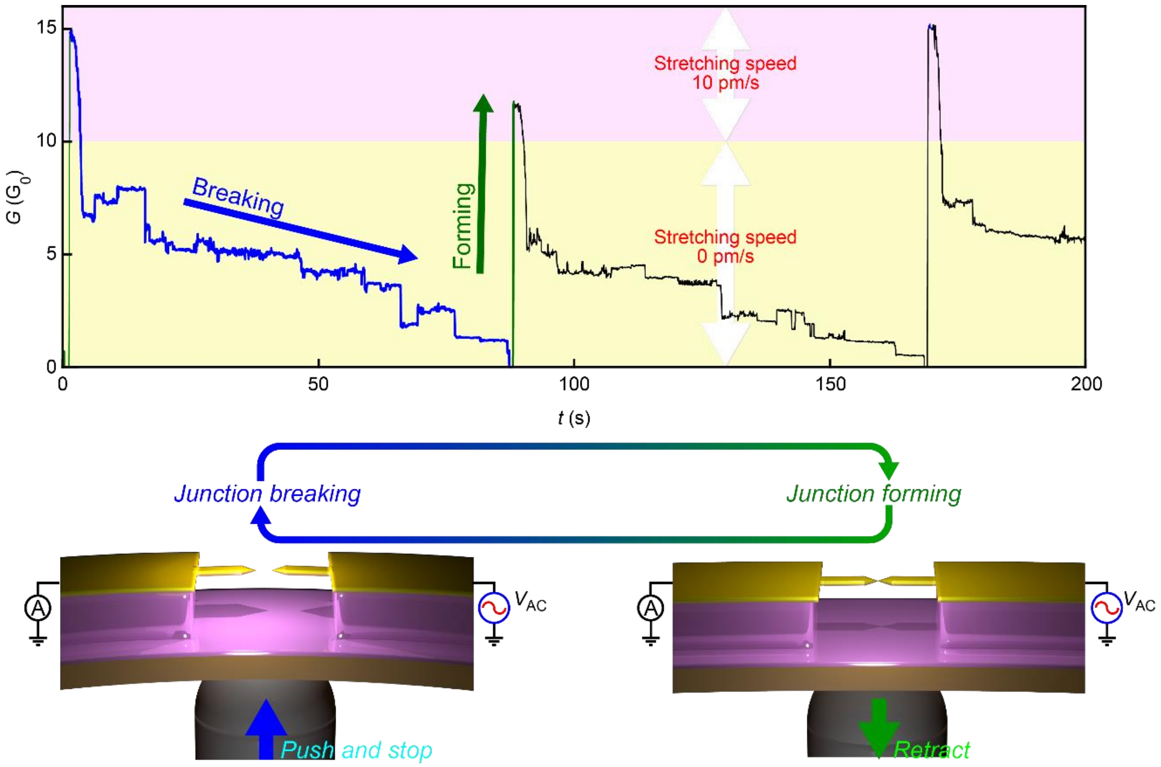

Figure S3. Break junction measurements under AC applied voltage. Sinusoidal voltage of 1 MHz frequency and amplitude of V_{pp} was biased to a Au junction. By bending the phosphor-bronze substrate through moving the pushing rod via piezo-actuator motion control, the junction was slowly elongated at room temperature in vacuum. The junction conductance G was

simultaneously monitored that tended to decrease in a step-wise fashion during the bending (blue curve in the top graph). When G < 10 G_0, we stopped the piezo motion and let the junction break spontaneously by thermal energy.[S2] Note that AC components were smeared to zero by the relatively long integration time used for the current sampling. When the conductance decreased to zero, which signifies breakdown of the Au junction, we swiftly move the pushing rod to release the bending force so as to reconnect the junction until G increased above 10 G_0 (green curve in the top graph). The series of the processes were repeated for 1000 times at each V_{pp} condition measured.

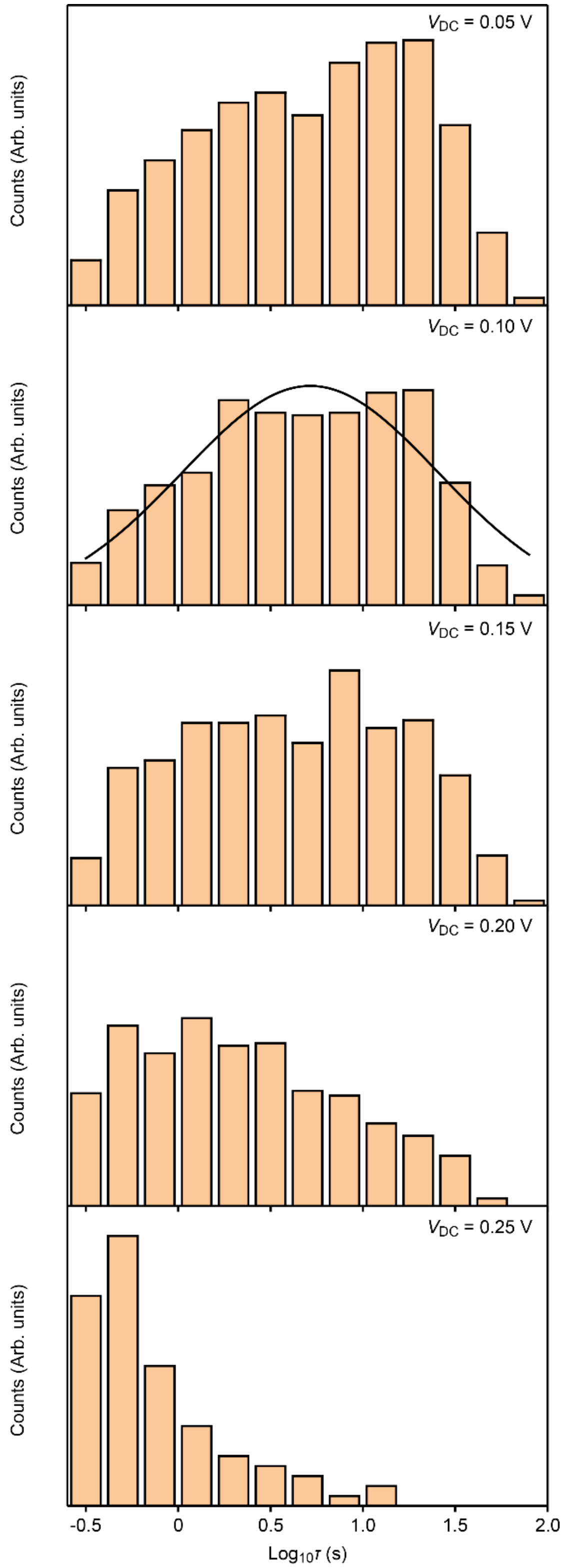

Figure S4. $\mathrm{Log}_{10}\tau$ histograms obtained under various DC voltage conditions V_{DC} ranging from 0.05 V to 0.25 V. Bin size is 0.2.

Supplementary references

S1. M. Tsutsui, M. Taniguchi, and T. Kawai. Formation and self-breaking mechanism of stable atom-sized junctions. *Nano Lett.* **8**, 345-349 (2008).

S2. Z. Huang, F. Chen, P. A. Bennett, and Nongjian Tao, Single molecule junctions formed via Au-thiol contact: Stability and breakdown mechanism. *J. Am. Chem. Soc.* **129**, 13225-13231 (2007).
